# An improved assay to measure the phospholipid transfer activity of microsomal triglyceride transport protein

**DOI:** 10.1016/j.jlr.2021.100136

**Published:** 2021-10-18

**Authors:** Narasimha Anaganti, Sujith Rajan, M. Mahmood Hussain

**Affiliations:** 1 Department of Foundations of Medicine, New York University Long Island School of Medicine, Mineola, NY, USA; 2 VA New York Harbor Healthcare System, Brooklyn, NY, USA

**Keywords:** triacylglycerol, fluorescence, lipid transfer, vesicles, NBD-TAG, dioleolylphosphoethanolamine, palmitoyloleoylphosphoethanolamine, saturation kinetics, lomitapide, high-throughput screen, apoB, apolipoprotein B, AV, acceptor vesicle, cDNA, complementary DNA, CV, coefficient of variation, DMPE, 1,2-dimyristoyl-*sn*-glycero-3-phosphoethanolamine, dMTP, *Drosophila* MTP, DOPE, 1,2-dioleoyl-*sn*-glycero-3-phosphoethanolamine, DPPE, 1,2-dipalmitoyl-*sn*-glycero-3-phosphoethanolamine, triethylammonium salt, DV, donor vesicle, hMTP, human MTP, MTP, microsomal triglyceride transfer protein, NBD, *N*-7-nitro-2-1,3-benzoxadiazol-4-yl, PE, phosphatidylethanolamine, PL, phospholipid, POPE, 1-palmitoyl, 2-oleoyl-*sn*-phosphoethanolamine, TAG, triacylglycerol

## Abstract

Microsomal triglyceride transfer protein (MTP) is essential for the assembly and secretion of apolipoprotein B-containing lipoproteins. MTP transfers diverse lipids such as triacylglycerol (TAG) and phospholipids (PLs) between vesicles in vitro. Previously, we described methods to measure these transfer activities using *N*-7-nitro-2-1,3-benzoxadiazol-4-yl (NBD)-labeled lipids. The NBD-TAG transfer assay is sensitive and can measure MTP activity in cell and tissue homogenates. In contrast, the NBD-PL transfer assay shows high background and is less sensitive; therefore, purified MTP is required to measure its PL transfer activity. Here, we optimized the assay to measure also the PL transfer activity of MTP in cell and tissue homogenates. We found that donor vesicles containing dioleoylphosphoethanolamine and palmitoyloleoylphosphoethanolamine result in a low background signal and are suitable to assay the PL transfer activity of MTP. This assay was capable of measuring protein-dependent and substrate-dependent saturation kinetics. Furthermore, the MTP inhibitor lomitapide blocked this transfer activity. One drawback of the PL transfer assay is that it is less sensitive at physiological temperature than at room temperature, and it requires longer incubation times than the TAG transfer assay. Nevertheless, this significantly improved sensitive assay is simple and easy to perform, involves few steps, can be conducted at room temperature, and is suitable for high-throughput screening to identify inhibitors. This assay can be adapted to measure other PL transfer proteins and to address biological and physiological importance of these activities.

The microsomal triglyceride transfer protein (MTP) is a heterodimer consisting of MTP (α) and protein disulphide isomerase (β) subunits (1, 2, 3) that transfers several lipids in vitro (4, 5, 6, 7, 8). This lipid transfer activity plays a major role in the lipidation of newly synthesized apolipoprotein B (apoB), and in the assembly of chylomicrons and VLDL, and their subsequent secretion by the intestine and liver, respectively (1, 9). Loss of the lipid transfer function of MTP because of mutations in the human *MTTP* gene causes abetalipoproteinemia, a disease condition associated with an absence of plasma apoB-containing lipoproteins (10, 11). Tissue-specific ablation of MTP decreases plasma lipids and lipoproteins in mice (7, 12). Moreover, antagonists that inhibit the lipid transfer activity of MTP decrease the assembly and secretion of apoB-containing lipoproteins, as well as plasma lipid levels (13, 14, 15). One inhibitor, lomitapide, has been approved for the treatment of familial hypercholesterolemia, but it is associated with hepatic steatosis (14, 16). Thus, the lipid transfer function of MTP is essential for lipoprotein assembly and plasma lipid homeostasis; consequently, easy methods to measure this activity are needed.

MTP transfers diverse classes of lipids, such as triacylglycerols (TAGs), cholesteryl esters, phospholipids (PLs), and sphingolipids, and it has been suggested to recognize hydrophobic motifs rather than individual lipids (5, 6, 7). Kinetic, structural, and mutational evidence supports the possible presence of at least two domains that might be involved in the transfer of neutral lipids, such as TAG, and charged lipids, such as PL (1, 3, 5, 17, 18). These two transfer activities have differential effects on apoB-containing lipoprotein assembly and secretion. First, MTP has been shown to have evolved as a PL transfer protein in invertebrates and to have acquired TAG transfer activity during vertebrate evolution (19). PL transfer has been demonstrated to be sufficient for the assembly and secretion of apoB-containing lipoproteins (20). Khatun *et al.* (21) have shown that the liver-specific expression of *Drosophila* MTP (dMTP) that only transfers PL in liver-specific MTP knockout (L-*Mttp*^*−/−*^) decreases plasma and hepatic lipids. Recently, Wilson *et al.* (18) have shown that zebrafish lacking the TAG transfer activity of MTP show no defects in growth and do not exhibit steatosis. Thus, targeted inhibition of the TAG transfer activity of MTP is likely to decrease plasma lipids without causing steatosis. To identify compounds that might differentially inhibit MTP activity, simple and efficient methods are needed to measure both the neutral and charged lipid transfer activities of MTP.

The MTP lipid transfer assays consist of two types of lipid vesicles: donor vesicles (DVs) and acceptor vesicles (AVs). The classical multistep assays use DVs containing radiolabeled lipids (4, 8). These vesicles are incubated with AVs that do not contain labeled lipids as well as a source of MTP. After incubation, DVs are separated from the reaction mixture, and the amounts of lipids transferred to AVs are quantified (4, 6, 8, 22). Because the handling and disposal of radioactive material is hazardous, fluorescence-based assays were developed, in which *N*-7-nitro-2-1,3-benzoxadiazol-4-yl (NBD)-labeled lipids replace the radioactive lipids (23, 24). These assays were based on the principle that two or more fluorescently labeled lipid monomers located in proximity in lipid vesicles form a nonemissive trap site, thus self-quenching their fluorescence (25). In these assays, fluorescently labeled lipids are incorporated in DV, thus resulting in the self-quenching of the fluorophore (23, 24). When MTP transfers lipids from DV to AV, the fluorescent lipid molecules are unquenched, thereby increasing the fluorescence representative of transfer activity. An advantage of these assays is that they do not require the separation of DV from AV. However, a disadvantage of these assays is their high background values, owing to the presence of unquenched lipids. Packaging of NBD-TAG in phosphatidylcholine (PC) vesicles results in a background less than 10% of total fluorescence (23). In contrast, incorporation of NBD-phosphatidylethanolamine (PE) in DV is associated with higher background and less stable DV (24). Therefore, the MTP PL transfer assay requires improvement.

The higher background after the incorporation of NBD-PE rather than NBD-TAG in PC vesicles probably results from PLs diffusing more easily than TAG from vesicles as soluble monomers. The rate of diffusion of PLs depends on their molecular structure as well as the characteristics of the donor and AVs (25). Furthermore, acyl chain length and the degree of saturation affect the diffusion and background fluorescence (25). Therefore, to decrease the background fluorescence, and to improve the sensitivity and reproducibility of the assay, we compared several NBD-PEs differing in their unlabeled fatty acid composition. We found that DV containing NBD-1-palmitoyl, 2-oleoyl-*sn*-phosphoethanolamine (POPE) and NBD-1,2-dioleoyl-*sn*-glycero-3-phosphoethanolamine (DOPE) showed high fluorescence quenching and a greater percentage transfer of of PL by human MTP (hMTP). The addition of unlabeled PE to these vesicles further increased the stability and reproducibility of the assay. This assay can successfully measure the PL transfer activity of MTP in cell and tissue homogenates.

## Materials and methods

### Materials

PC (no. 131601C) and fluorescently labeled ammonium salts of PEs with the acyl groups, NBD-1,2-dimyristoyl-*sn*-glycero-3-phosphoethanolamine (DMPE; no. 810143C), NBD-POPE (no. 810118P), or NBD-DOPE (no. 810145C) were obtained from Avanti Polar Lipids (Alabaster, AL). NBD-1,2-dipalmitoyl-*sn*-glycero-3-phosphoethanolamine, triethylammonium salt (DPPE; no. N360) was obtained from Invitrogen (Thermo Fisher Scientific). Lomitapide, an MTP inhibitor, was obtained from Sigma (no. SML1385-5MG). The Cos-7 and Huh-7 cell lines, and the plasmids pcDNA3 and pcDNA3-hMTP-FLAG, have been described previously (20, 21, 26, 27). Anti-FLAG® M2 affinity agarose gel and other chemicals were purchased from Millipore Sigma (no. A2220-5ML, St. Louis, MO). Round bottom black 96-well assay plates were obtained from Costar (no. 3792; Kennebunk, ME). FLAG peptide was custom synthesized (GenScript).

### Acceptor and DV preparation

The AVs were prepared as before (23, 24). In brief, 92.3 μl of PC (32.5 mmol/ml stock in chloroform) was dried under nitrogen gas and then suspended in 1 ml of 15 mM Tris buffer, pH 7.4, containing 40 mM NaCl, 1 mM EDTA, and 0.02% NaN_3_, to achieve a final PC concentration of 3 mmol/ml. The suspension was sonicated on ice with 40% amplitude, 60 s pulse on and 5 s off, with a 550 Sonic Dismembrator (Thermo Fisher Scientific) until the turbid solution became clear. The sonicated vesicles were centrifuged at 60,000 rpm (150,000 *g*) in an Optima Max-TL Ultracentrifuge with a TLA110 rotor (Beckman Coulter) at 4°C for 1 h. Supernatants were collected, and 140 mg of NaCl and 20 mg of BSA were added. The vesicles were stored at 4°C. Similarly, DVs were prepared with different NBD-labeled PEs. Briefly, the mixture of PC at 0.4 mmol/ml (12.3 μl of 32.5 mmol/ml stock solution) and NBD-PE at 30, 60, 90, 120, 150, and 180 μmol/ml, with or without 90 μmol/ml cold PE, were used for the preparation of DV (1 ml). These vesicles were separately stored at 4°C.

### MTP expression, purification, and detection

The plasmid carrying the complementary DNA (cDNA) of the hMTP cDNA (pcDNA3-hMTP-FLAG) and the empty vector as a control were transfected into Cos-7 cells with EndoFectin Max (no. EF013; GeneCopoeia) as previously described (17, 20, 26, 27, 28). At 48 h after transfection, cells were collected in 1 ml buffer K (10 mM Tris–Cl, 1 mM MgCl_2_, and 1 mM EGTA, pH 7.4) containing 150 mM NaCl and lysed by sonication on ice with 35% amplitude, 2 s pulse on and 1 s off, for 90 s (550 Sonic Dismembrator; Thermo Fisher Scientific). The cell lysates were spun at 12,000 rpm or 13,500 *g* in a 5424R (Eppendorf) centrifuge for 10 min at 4°C to remove unbroken cells and cell debris. The protein concentration was estimated with a Pierce BCA protein assay kit (no. 23225; Thermo Fisher Scientific) and BSA standard.

The FLAG-tagged MTP was purified with columns packed with anti-FLAG M2 affinity agarose beads. We added 2 ml of anti-FLAG M2 affinity agarose beads to each column (no. KT420400-0730; Kontes Flex column) and allowed them to settle without air bubbles. The columns were then washed with 10 bed volumes of 10 mM Tris buffer, pH 7.4. The clear cell lysates were then applied to these columns. The columns were washed with 20 ml of 10 mM Tris buffer, pH 7.4, containing 150 mM NaCl. MTP was eluted with 10 ml of 100 μM FLAG peptide in the same wash buffer. The entire procedure was conducted at 4°C. The removal of FLAG peptide and concentration of the purified protein was done by ultrafiltration with a 3 kDa cutoff centrifugal filter (Amicon Ultra 15, no. UFC900324; Merck Millipore Ltd). First, the centrifugal filter was washed with 5 ml of ultrapure water by centrifugation (5,000 rpm at 4°C, Sorvall Lynx 4000 centrifuge with A27-8x50 rotor; Thermo Fisher Scientific). The eluted MTP was subsequently added to the filter and centrifuged until the 5 ml of solution was concentrated to 1 ml. Then 4 ml of 10 mM Tris buffer was added, and the solution was centrifuged again until a 0.5 ml volume containing pure MTP remained. The purified proteins along with crude proteins were resolved on an 8% SDS-PAGE gel and stained with Coomassie blue or were used for immunoblotting with anti-FLAG antibody (no. F3165; Sigma).

### Preparation of cell homogenates to assay MTP activity

MTP expressing Cos-7 cells and Huh-7 cells were washed three times with PBS; collected from the flasks or culture dishes in buffer K and lysed by sonication. The lysates were centrifuged at 12,000 rpm for 10 min, as detailed previously. The clear cell lysates were collected, and 50 μg of protein was used for MTP assays.

### Preparation of tissue homogenates to assay MTP activity

*Mttp*^*fl/fl*^ and liver-specific MTP knockout (L-*Mttp*^*−/−*^) mice were maintained in regular chow diet (PicoLab Rodent diet 20; no. 5053) at the NYU Langone School of Medical Science animal facility. Institutional Animal Care and Use Committee approved all experimental protocols. These mice were sacrificed under deep anesthesia at 10 AM by cervical dislocation, and transcardial perfusion was done with normal saline before dissecting different organs. The organs were snap frozen in liquid nitrogen and kept in −80°C until use. Approximately, 10 mg (liver and intestine) and 100 mg (epididymal white adipose tissue) was crushed with a mortar and pestle (Cole-Parmer PTFE Tissue Grinder; no. UX-44468-11) cooled with liquid nitrogen. The crushed tissues were suspended in 1 ml of buffer K containing 10 μl/ml of protease inhibitor cocktail (no. P2714; Sigma), homogenized with a Dounce homogenizer, and centrifuged at 12,000 rpm for 10 min at 4°C as described previously. The supernatant was collected and diluted appropriately for MTP assays.

### Measurement of the lipid transfer activity of MTP

The PL transfer activity of MTP was measured in both cell homogenates, and purified proteins obtained from Cos-7 cells were transfected with pcDNA plasmids for the expression of hMTP or control plasmids. In addition, Huh-7 human hepatoma cells and liver homogenates from *Mttp*^*fl/fl*^ and L-*Mttp*^*−/−*^ (7, 21) were used in the assays. The PL transfer activity was measured in triplicate with a mixture of 5 μl of AV (15 μmol PC) and 5 μl of DV (450 nmol NBD-PE and 2 μmol PC) in a total volume of 100 μl. First, the DV and AV were added to the wells of a 96-well black round bottom microtiter plate (Costar; no. 3792), and then 90 μl of sample containing either crude (50 μg) or purified (25 ng) protein was added. For blank controls, only buffer was added to the vesicles. For total fluorescence measurements, isopropanol (90 μl) was added to a mixture of DV and AV (10 μl), and the fluorescence was measured immediately. For MTP assays, DV and AV were incubated with a source of MTP. The fluorescence was measured at different time points with λ_ex_ of 463 nm and λ_em_ of 536 nm with an Enspire plate reader (PerkinElmer). For MTP inhibition study, samples containing MTP were incubated with various concentrations of lomitapide at room temperature for 10 min and mixed with DV and AV. Fluorescence measurements were recorded over a time course of 4 h. The TAG transfer activity of MTP was measured as previously described (23, 27).

### Statistics

The percentage quenching (%Q) was calculated with the formula (%Q = [Ft − Fb/Ft] × 100) (25), where Ft is the total fluorescence measured in the presence of isopropanol and Fb is the blank or background fluorescence in the presence of buffer. The percentage transfer (%T) of PL or TAG was calculated as previously described (23, 24) with the formula (%T = [Fs − Fb)/(Ft − Fb] × 100), where Fs is the fluorescence of the test sample, Fb is the fluorescence of the blank, and Ft is the total fluorescence. The intra-assay and interassay coefficient of variation (CV) was calculated with the standard formula CV = (SD/mean) × 100. All graphing and statistical analyses were performed in GraphPad Prism software, version 8.4.3 (GraphPad Software, Inc).

## Results

### Incorporation of different NBD-PEs in PC vesicles results in varying background fluorescence

**Fig. 1:**
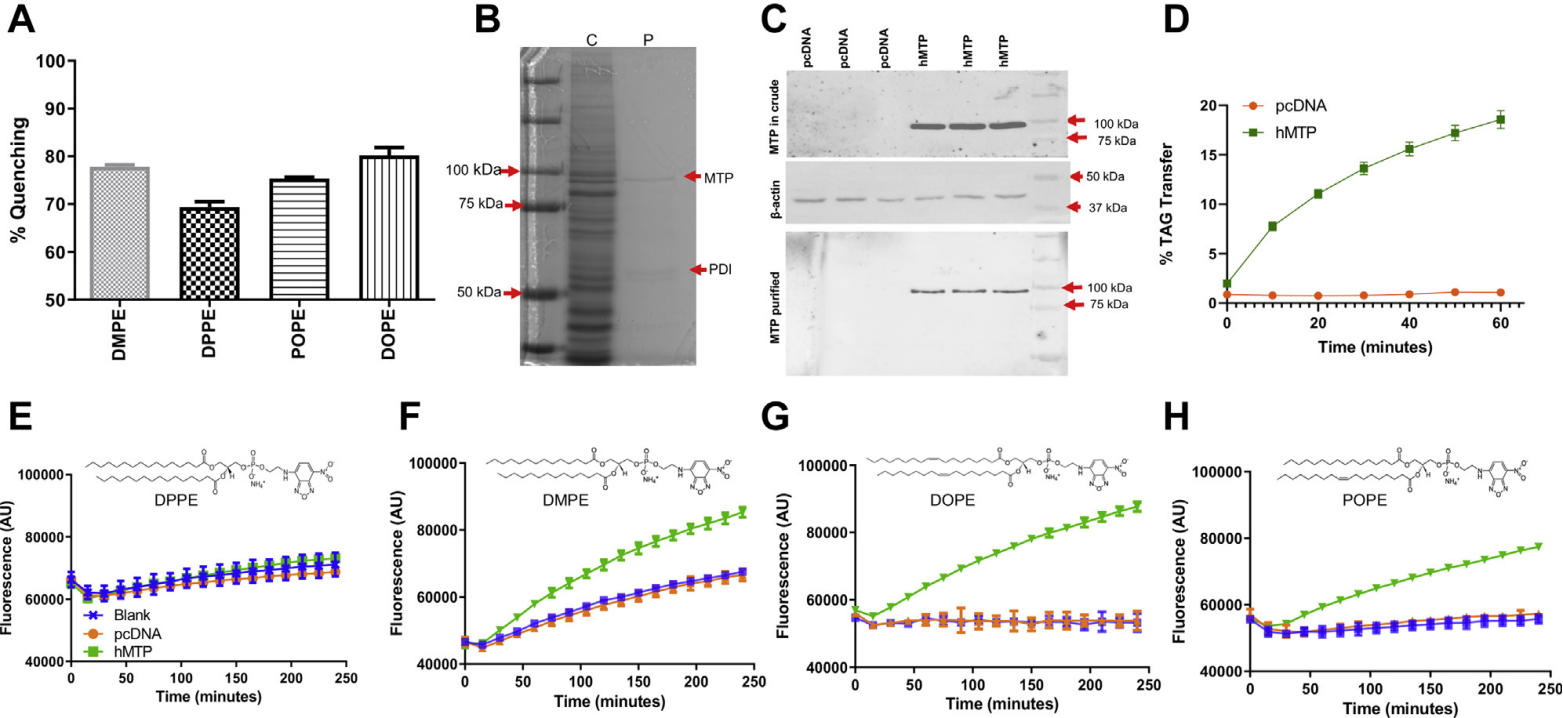
Evaluation of different NBD-PEs for their suitability to measure the PL transfer activity of MTPs. A: The percentage quenching of NBD fluorescence in DV made with different PEs. DVs to measure the PL transfer activity of MTPs were made with four different NBD-labeled PEs: *i*) DMPE, *ii*) DPPE, *iii*) POPE, and *iv*) DOPE. The percentage fluorescence quenching was calculated after measurement of the total fluorescence in the presence of isopropanol and of the blank in the presence of buffer. B and C: MTP was expressed in Cos-7 cells and purified from cell lysates with anti-FLAG M2 affinity gel. B: The crude (C) and purified (P) proteins were resolved on 8% SDS-PAGE and Coomassie stained. The MTP and PDI bands are visible on the gel in the purified fraction. C: For validation of the expression level and purification, both cell lysates (crude) and purified fractions were resolved on SDS-PAGE, transferred to a nitrocellulose membrane, and probed with anti-FLAG antibody (top and bottom). D: TAG transfer activity of purified MTP (25 ng). E–H: DVs made with (E) DPPE, (F) DMPE, (G) DOPE, and (H) POPE were used to measure the PL transfer activity of purified MTP in triplicate. The activity of purified hMTP was measured using a mixture of 5 μl of DV (0.45 μmol NBD-PE + 2 μmol PC) and 5 μl of AV (15 μmol PC). Fluorescence readings were recorded over time. Blank represents readings in buffer only. All PL transfer assays were performed at room temperature, and experiments were repeated at least three times with technical duplicates. The lines and error bars represent means ± SD. The error bars are plotted but are smaller than the symbols. PDI, protein disulphide isomerase.

The MTP lipid transfer assays using NBD-labeled compounds were based on the principle that incorporation of NBD lipids in lipid vesicles results in fluorescence quenching (23, 24, 29). During transfer, the NBD-lipids are unquenched, and the increases in fluorescence over time are recorded to quantify the transfer activity. Here, we first evaluated different NBD-PEs for their ability to be incorporated into PC vesicles by sonication (30), the extent of fluorescence quenching and the remaining unquenched background fluorescence, and the ability of purified hMTP to transfer these NBD-PEs. All NBD-PEs were incorporated into PC vesicles via sonication, as evidenced by the turbid mixture becoming clear after sonication. This finding is consistent with those of studies showing that PE does not form bilayers but can be incorporated into PC bilayers (31). First, we determined the %Q and percentage background (i.e., the fluorescence in the presence of buffer) in DV. DVs were dissolved in isopropanol to measure the total fluorescence. The %Q for different NBD-PEs ranged between 70% and 80% (Fig. 1A). Therefore, 20–30% of the total fluorescence remained unquenched as background in these vesicles. The highest quenching (>80%) was observed for DOPE-containing DV.

Next, we evaluated whether these vesicles were suitable to measure PL transfer activity of MTPs. Cell and tissue homogenates contain other PL transfer proteins, for example, PL transfer protein, phosphatidylinositol transfer protein, and phosphatidylcholine transfer protein, which may interfere with the measurement of MTP activity (32, 33, 34). To avoid interference with these proteins, we first used purified hMTP. For purification, we expressed FLAG-tagged hMTP in Cos-7 cells and performed purification with anti-FLAG M2-affinity gel as previously described (17, 23, 24, 26, 27). The large MTP and small protein disulphide isomerase bands were visible in the purified fraction by Coomassie staining (Fig. 1B). Western blotting with anti-FLAG antibodies was used to further characterize the expression and purification of MTP. The MTP-FLAG chimera was detected in cell lysates and purified fractions (Fig. 1C). As a loading control, β-actin (middle blot) was used. We then used the purified MTP to determine the TAG transfer activity. The purified MTP showed robust TAG transfer activity (Fig. 1D). Thus, the purified hMTP chimera was recognized by antibodies and was able to transfer TAG.

We then used this purified MTP to measure PL transfer activity. DVs containing different NBD-PEs were incubated with AV and purified MTP as test samples (Fig. 1E–H). For a negative control, we used Cos-7 cells transfected with pcDNA3 only. For the blank control, DV and AV were incubated with no protein. No differences among the blank, test sample, and negative control fluorescence values were observed when assays were performed with DV containing NBD-DPPE (Fig. 1E), thus indicating that these DVs are not suitable for measuring the PL transfer activity of MTP. However, the increases in fluorescence over time were significantly higher when DVs containing NBD-DMPE were used in test samples (Fig. 1F). In these conditions, the fluorescence values also increased over time in the blank and negative controls (Fig. 1F), thus perhaps indicating the instability of vesicles during incubation. These increases in the negative control and blank values may be problematic when MTP mutants with diminished PL activity are measured. Test samples with NBD-DOPE DV showed significantly greater fluorescence than blank and pcDNA3 negative controls (Fig. 1G). Similarly, the POPE-containing DV showed no significant fluorescence change over time in the blank and control wells. However, we observed a significant increase in fluorescence over time in test samples containing purified MTP (Fig. 1H). These results suggested that DVs with NBD-POPE and NBD-DOPE are useful to measure the PL transfer activity of MTP. Although both POPE and DOPE showed no changes in blank and control fluorescence over time, the DV containing NBD-DOPE had a higher %Q and less background and therefore were used in further experiments.

### NBD PE concentration influences the PL transfer assay

**Fig. 2:**
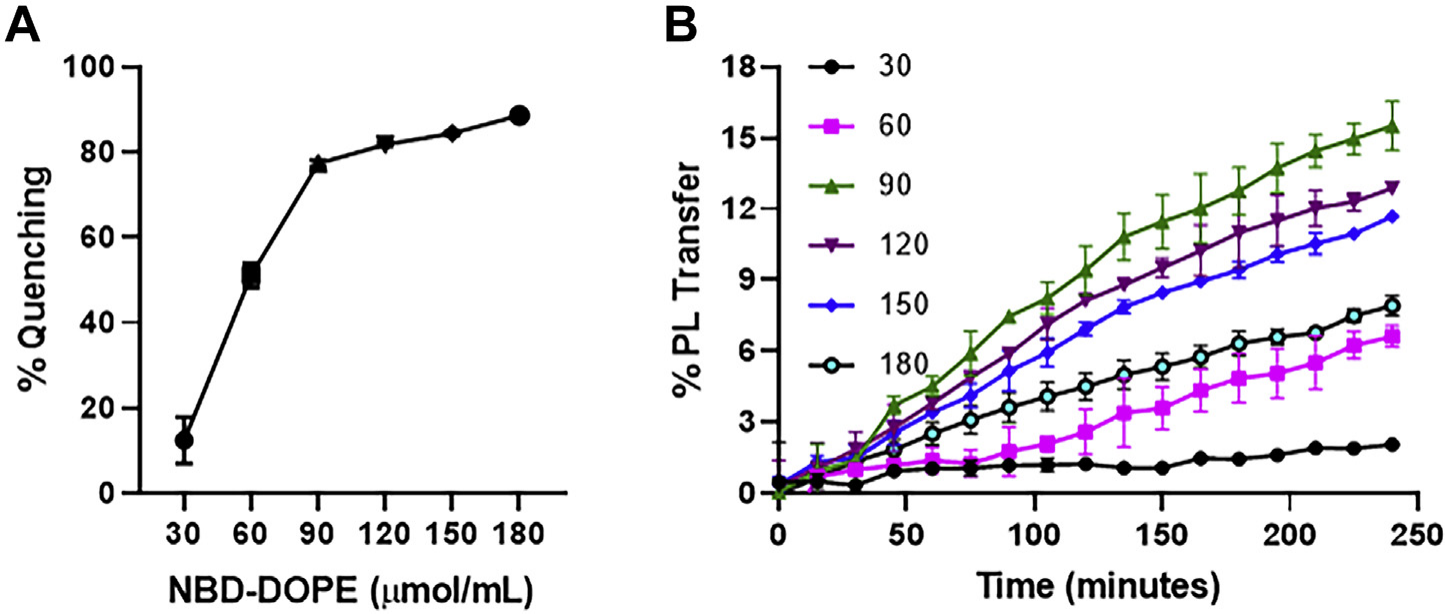
Determination of optimal NBD-DOPE amounts for lipid transfer activity. A: For identification of the optimum concentration of NBD-DOPE, increasing concentrations of NBD-DOPE (30–180 μmol) were mixed with 400 μmol of PC during vesicle preparation. Fluorescence quenching increased linearly with increasing concentrations up to 90 μmol of NBD-DOPE. Further increases in NBD-DOPE amounts did not significantly increase quenching. Data are representative of three experiments. Assays were performed in triplicate. Lines connect means, and error bars represent the SD. The error bars are plotted but are smaller than the symbols. B: Purified hMTP was incubated with DV containing different amounts of NBD-DOPE (30–180 μmol/ml). The percentage transfer of PL was measured over time. The highest PL transfer was observed with DV containing NBD-DOPE at 90 μmol/ml. Data are representative of three independent experiments. The lines and error bars represent means ± SD.

In the next step, we aimed to determine the optimum concentration of NBD-DOPE incorporated in DV to achieve maximum lipid transfer activity. To do so, we constructed DVs with different concentrations (30–180 μmol/ml) of NBD-DOPE with fixed amounts of PC (∼400 μmol/ml), thereby changing the PC:PE ratios from 1:13 to 1:2. As the concentration of NBD-DOPE increased, the fluorescence quenching also increased and reached a maximum 80%Q at 90 μmol/ml (Fig. 2A). These DVs containing different concentrations of NBD-DOPE were then used to assess the MTP transfer activity with fixed amounts of AV. The highest lipid transfer was observed with DV made with 90 μmol/ml NBD-DOPE (Fig. 2B, green). These results suggested that 90 μmol/ml NBD-DOPE is optimal for producing DV to achieve the highest quenching and percentage transfer of PL.

### Stability of DVs

**Fig. 3:**
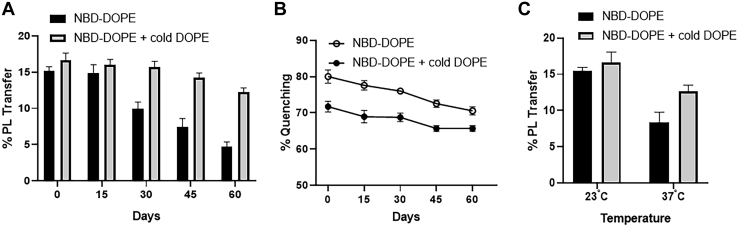
Stability of DVs. To increase the stability of DV, we added unlabeled DOPE along with NBD (NBD-DOPE + cold DOPE) and compared them with DV containing only NBD-DOPE. A and B: The stability at 4°C of two different DVs was assessed for a period of 60 days by measuring (A) the percentage transfer of PL by purified hMTP and (B) the percentage fluorescence quenching. C: Temperature sensitivity of the DV was assessed by measurement of the PL transfer activity of MTP at room temperature and 37°C. All experiments were repeated at least three times and in technical duplicate. The error bars represent SD.

We then studied the stability of NBD-DOPE vesicles stored at 4°C by assaying the PL transfer activity of MTPs at different times (Fig. 3A). These vesicles were stable for 15 days, on the basis of an absence of changes in the transfer activity; however, after longer storage times, MTP was less efficient in transferring NBD-DOPE (Fig. 3A), thereby indicating that these vesicles were not suitable for use in MTP assays after 15 days. We reasoned that the decreased activity might be due to rearrangement of lipid packing in the vesicles. Therefore, we measured changes in %Q and observed that the quenching also declined over time (Fig. 3B). The loss of quenching over a period of 60 days suggested possible destabilization of vesicles, perhaps by collision and/or gradual diffusion of NBD-PE from the DVs (35). Therefore, we attempted to stabilize these vesicles.

The effects of different amounts of PE in PC bilayers have been studied (31). The equilibrium properties of mixed PC/PE bilayers are known to be a function of PC/PE composition. At lower PE content, PC forms mixed bilayers. The area of the bilayer, order parameters of lipid tails, and lateral mobility of lipids are all linearly related to the PE concentration, and they level off as the PC:PE ratio in vesicles reaches 1:3 (31). In our DV, we used a PC:PE ratio of 4:1. Therefore, we reasoned that increasing the amounts of PE might stabilize the vesicles. To test whether the addition of unlabeled DOPE might increase the stability of DV, we included an amount of cold DOPE (90 μmol/ml) equal to that of NBD-PE during vesicle formation, to obtain a PC:PE ratio of 2:1. The addition of unlabeled (cold) DOPE resulted in 8% lower quenching than that was observed with vesicles made without unlabeled PE on day 0. Nevertheless, the decrease in quenching over time was less steep in DV composed of NBD-DOPE + cold DOPE DV than vesicles without unlabeled PE (Fig. 3B). More importantly, these vesicles yielded similar percentage transfer activity for 45 days and were still suitable for use until day 60 (Fig. 3A). Thus, the addition of unlabeled PE increases background as well as stability, thereby allowing the vesicles to be used in assays after longer storage periods. The small increases in background did not appear to have significant adverse effects on the measurements of the PL transfer activity of MTPs.

Next, we assessed the effect of temperature by performing PL transfer assays at room temperature (23°C) and at 37°C. The percentage transfer of PL by MTP with DV either with or without cold DOPE was higher at room temperature than at 37°C (Fig. 3C). Because lipid fluidity increases with temperature (36), these vesicles may become destabilized at 37°C, thereby increasing the background fluorescence and becoming less efficient in measuring the lipid transfer activity of MTPs. MTP transfers TAG at room temperature (23, 24). These data suggest that using DV containing NBD-DOPE + cold DOPE with a PC:PE ratio of 2:1 can efficiently measure PL transfer activity of MTP at room temperature.

### Effects of different protein and substrate concentrations on the PL transfer activity of MTPs and its inhibition by lomitapide

**Fig. 4:**
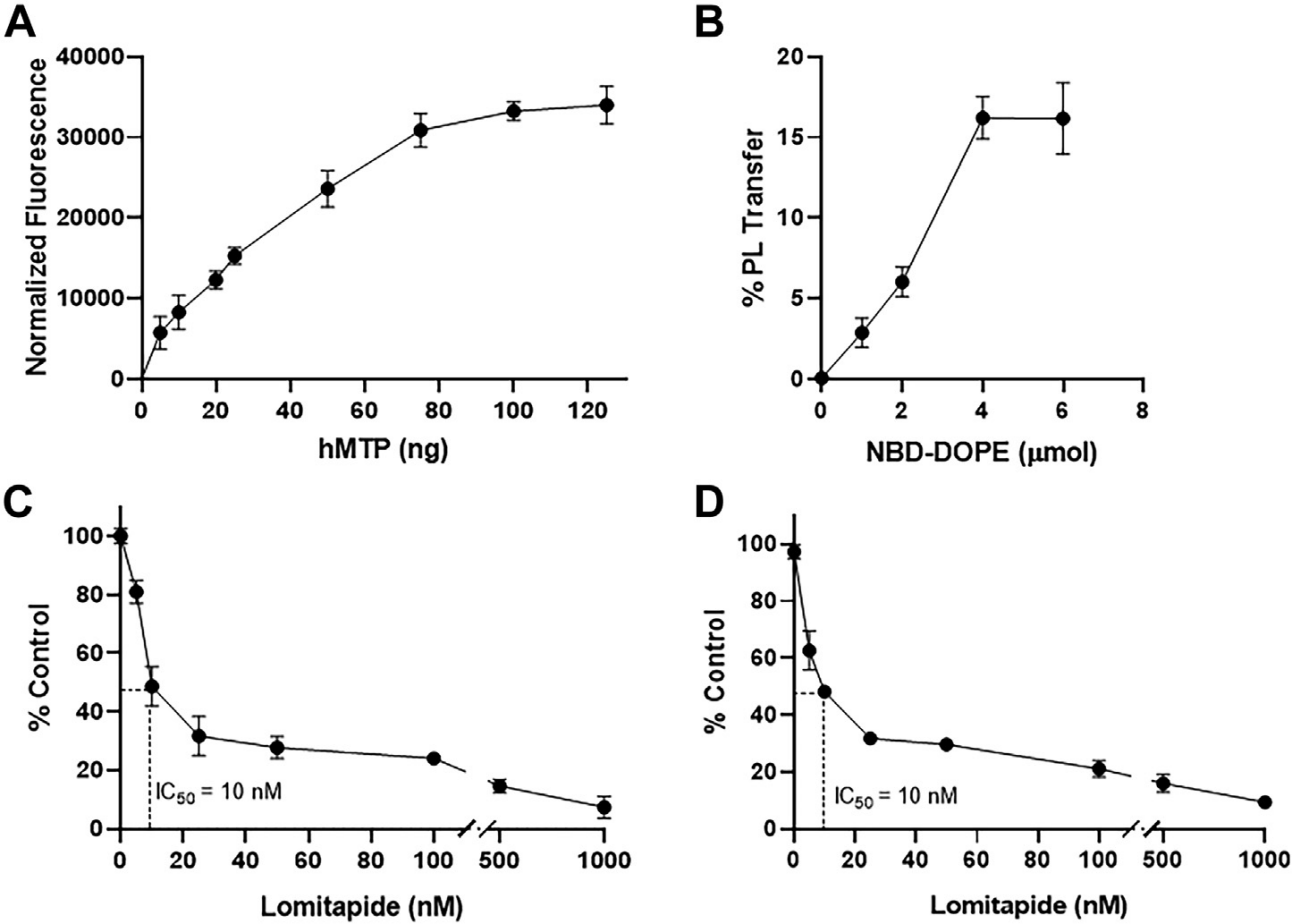
Protein and substrate concentration-dependent changes in the PL transfer activity of hMTP and the inhibition of this activity by lomitapide. A and B: To determine whether PL transfer increases with increasing concentrations of hMTP and DV, we performed PL transfer assays with DV made with NBD-DOPE and cold DOPE, as in Fig. 3. A: The activity increased with increasing protein concentrations until 80 ng and reached saturation at higher concentrations. B: The activity also increased with increasing concentrations of NBD-DOPE up to 4 μmol. C and D: Effects of lomitapide on the PL and TAG transfer activity of MTP. Purified MTP (25 ng) was incubated with various concentrations of lomitapide for 10 min at room temperature, and the assay was performed at room temperature to study the effects of different inhibitor concentrations on the (C) PL and (D) TAG transfer activity. Fifty percent inhibition (IC_50_) for both PL and TAG transfer activity was observed with 10 nM of lomitapide.

In PL transfer assays performed with increasing concentrations of purified hMTP (0–100 ng), we observed an initial increase in PL transfer activity with increasing protein concentrations that reached saturation at approximately 80 ng of hMTP (Fig. 4A). Similarly, the PL transfer increased with increasing amounts of substrate up to 4 μmol of NBD-DOPE and plateaued thereafter (Fig. 4B). To understand the effect of an MTP inhibitor on the PL transfer activity, we used the MTP antagonist lomitapide (16, 37). Increased inhibition of MTP activity was observed with increasing concentrations of lomitapide, with an IC_50_ value of 10 nM (Fig. 4C). The results corroborated those of earlier reports that MTP antagonists inhibit the PL transfer activity of MTP (24). Next, we determined the IC_50_ for the inhibition of the TAG transfer activity of MTP. Again, 10 nM lomitapide inhibited 50% of the TAG transfer activity (Fig. 4D). Thus, this inhibitor is equipotent in inhibiting the PL and TAG transfer activity of MTP.

### Measuring the PL transfer activity of MTPs in cells

**Fig. 5:**
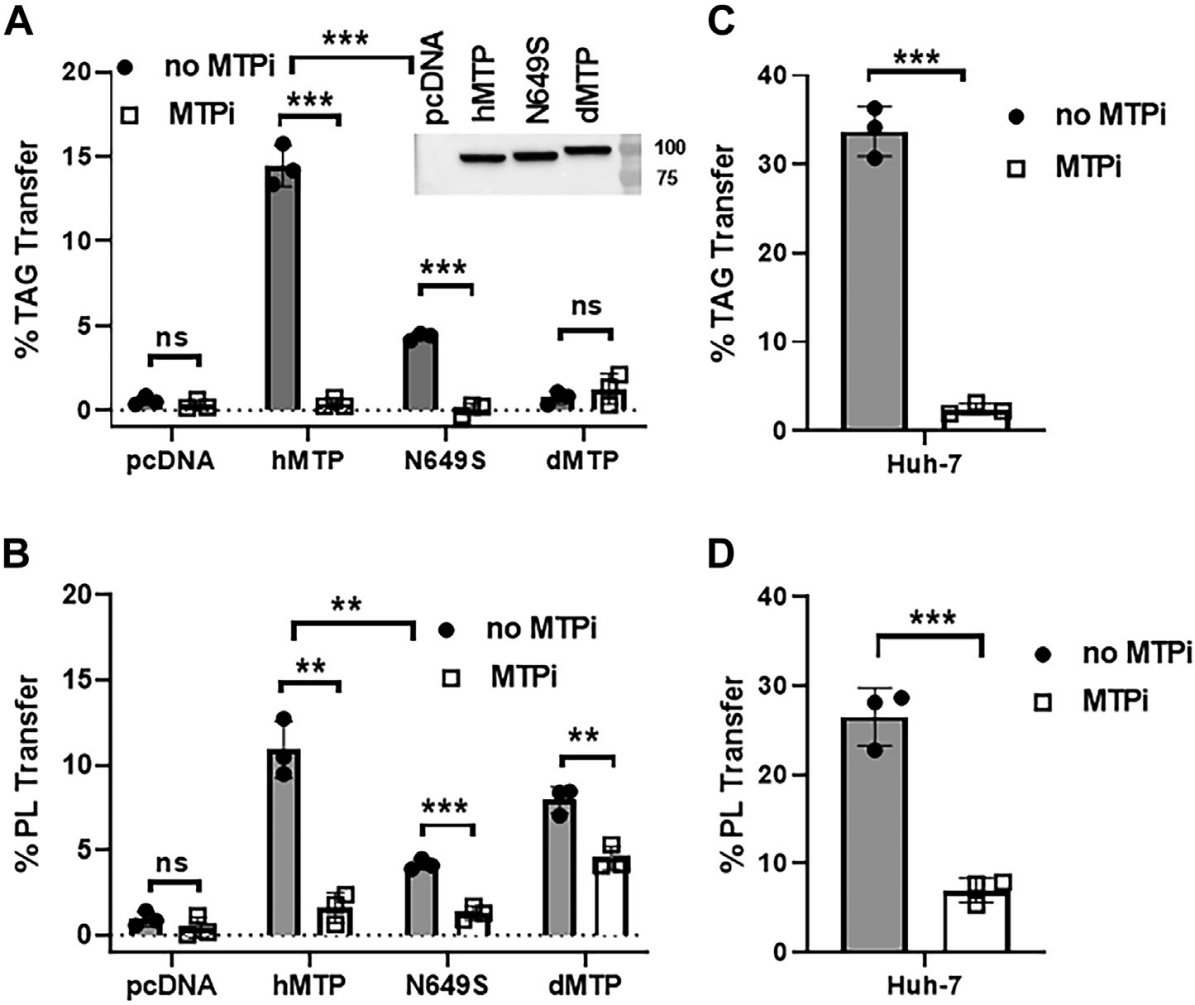
PL transfer activity of MTP in cells. A and B: hMTP, the N649S mutant, and dMTP were expressed in Cos-7 cells. Cells were lysed by sonication, and clear lysates were used for the assays. The Western blot images show comparable expression of three MTPs. Transfer activity of (A) TAG and (B) PL in the absence or the presence of lomitapide (1 μM). The cell lysates from pcDNA3 transfected cells served as negative controls. Results are representative of three independent experiments. All analyses were performed in technical duplicate. Unpaired *t*-tests were used to determine the significance of differences between samples with or without lomitapide treatment and between wild-type and MTP mutant. The error bars represent means ± SD. C and D: Huh-7 human hepatoma cell lysates were prepared as described in the *Materials and Methods* section and used to assay the transfer activity of (C) TAG and (D) PL in the presence or the absence of lomitapide. The data are representative of three independent experiments. Error bars represent SD, ∗*P* < 0.05, ∗∗*P* < 0.01, and ∗∗∗*P* < 0.001 as calculated by unpaired Student's *t*-test for graph.

To test whether these NBD-DOPE-containing DV could be used to measure the PL transfer activity of MTPs in cell homogenates, we performed PL transfer assays in Cos-7 cell homogenates expressing wild-type hMTP, N649S MTP mutant (38), or dMTP, which transfers only PL (20), in the presence or the absence of lomitapide. As a control, we also measured TAG transfer activity. Expression of hMTP resulted in significantly higher TAG transfer activity than that in pcDNA transfected cells (Fig. 5A). This activity was completely inhibited by lomitapide (Fig. 5A). Expression of hMTP also significantly increased the PL transfer activity in Cos-7 cells that was inhibited by lomitapide (Fig. 5B). Expression of the N649S MTP mutant, which is associated with abetalipoproteinemia (38), resulted in significantly lower TAG transfer activity, which was completely inhibited by lomitapide (Fig. 5A). Similar analyses for the PL transfer activity revealed that this mutant had significantly less PL transfer activity than that of wild-type hMTP and was inhibited by lomitapide (Fig. 5B). These data indicated that the PL transfer assay can detect relatively low levels of MTP activity.

We also used this assay to measure the PL transfer activity of dMTP. As we previously reported (20, 21), dMTP showed no TAG transfer activity (Fig. 5A). However, dMTP showed substantial PL transfer activity, which was slightly lower than that of hMTP (Fig. 5B). This activity was partially inhibited by lomitapide. Our findings suggest that this assay could be used to measure the PL transfer activity of dMTP in cell lysates. Moreover, these studies show that lomitapide is a less potent inhibitor of dMTP than hMTP.

The aforementioned results indicated that our assay can successfully measure the PL transfer activity of different MTPs after their overexpression in cells that do not express endogenous MTP. We next asked whether this assay could be used to measure the endogenous PL transfer activity of MTP in cells expressing MTP. For this purpose, we measured the PL transfer activity of MTPs in Huh-7 human hepatoma cells (Fig. 5C, D). The Huh-7 cells exhibited robust TAG transfer that was inhibited >95% by lomitapide (Fig. 5C). Similarly, the new optimized assay detected robust PL transfer activity in these cells, and lomitapide inhibited this activity (Fig. 5D). The observation of less inhibition of PL transfer activity in Huh-7 cells than MTP activity in Cos-7 cells likely indicates the presence of other PL transfer proteins in Huh-7 cells.

### Measuring the PL transfer activity of MTPs in tissue homogenates

**Fig. 6:**
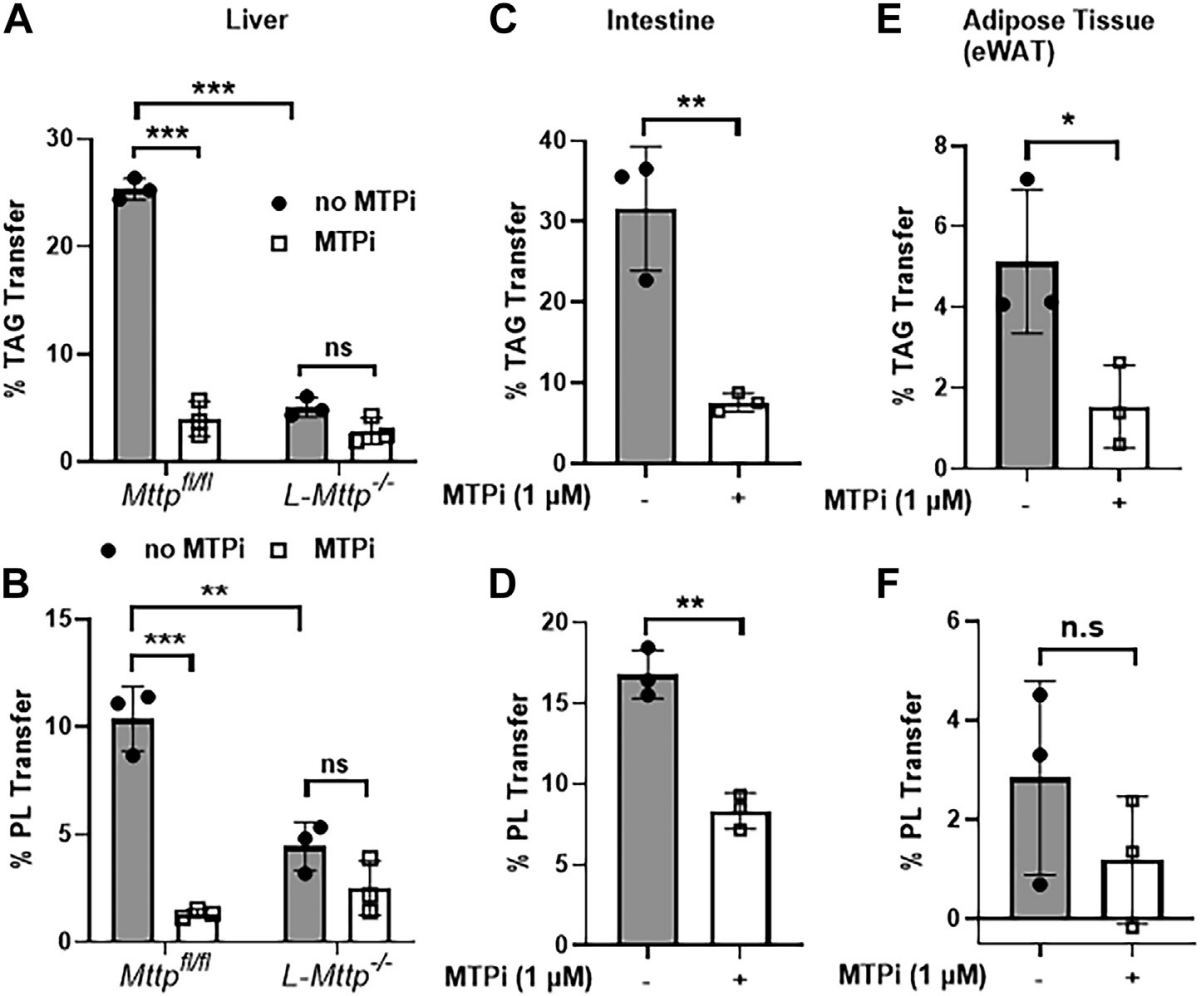
PL transfer activity of MTP in tissue homogenates. *Mttp*^*fl/fl*^ and L-*Mttp*^*−/−*^ mice (n = 3) were sacrificed at morning 10 AM, and organs were collected after transcardial perfusion with saline. A and B: Liver homogenates (50 μg) were used to measure (A) TAG transfer activity and (B) PL transfer activity in the presence and the absence of MTP inhibitor (1 μM lomitapide). C and D: Intestinal homogenate (50 μg) from mMTP was used to measure the (C) TAG transfer activity and (D) PL transfer activity in the presence and absence of MTP inhibitor (1 μM lomitapide). E and F: Epididymal white adipose tissue homogenates (50 μg) from *Mttp*^*fl/fl*^ were used to measure the (E) TAG transfer activity and (F) PL transfer activity in the presence and absence of MTP inhibitor (1 μM lomitapide). Error bars represent SD, ∗*P* < 0.05, ∗∗*P* < 0.01, and ∗∗∗*P* < 0.001 as calculated by one-way ANOVA followed by multiple comparison for graph (A), (B), unpaired Student's *t*-test for graph (C–F).

Next, we asked whether this assay could detect the PL transfer activity of MTPs in mouse liver. To evaluate this possibility, we used liver homogenates from *Mttp*^*fl/fl*^ and L-*Mttp*^*−/−*^ mice (7). As expected, we observed substantial TAG transfer activity in *Mttp*^*fl/fl*^ liver tissue, and this activity was inhibited by lomitapide (Fig. 6A). The TAG transfer activity was very low in liver homogenates of L-*Mttp*^*−/−*^ mice, and the residual activity was resilient to lomitapide inhibition (Fig. 6A). We then measured PL transfer activity in these liver homogenates (Fig. 6B). Substantial PL transfer was detected in the *Mttp*^*fl/fl*^ liver homogenates. Moreover, lomitapide significantly inhibited this activity. Liver homogenates from L-*Mttp*^*−/−*^ mice had a PL transfer activity 60% lower than that observed in *Mttp*^*fl/fl*^ livers. The residual PL transfer activity in L-*Mttp*^*−/−*^ mice was not inhibited by lomitapide. These studies indicated that our assay could successfully detect the PL transfer activity of MTPs in cell and tissue homogenates.

Based on the evidence that lomitapide completely inhibits the TAG transfer activity of MTPs in Cos-7 cells (Fig. 6A), we calculated the TAG transfer activity attributable to MTP in Huh-7 cells and liver homogenates. In Huh-7 cells and mouse livers, greater than 80–90% of the TAG transfer activity was attributable to MTP. Similar analyses of PL transfer activity revealed that MTP contributes to 70–80% of the PL transfer activity in Huh-7 cells and mouse livers. Thus, MTP is the major transfer protein involved in TAG and PL transfer in the liver.

We then studied MTP activity in the intestine. We observed substantial TAG and PL transfer activity in intestinal homogenates (Fig. 6C, D). Lomitapide inhibited almost 75% of the TAG transfer activity (Fig. 6C). In contrast, about 60% of the PL transfer activity was inhibited in the intestine (Fig. 6C). Since MTP is also expressed in the adipose tissue, we measured activity in epididymal white adipose tissue homogenates of *Mttp*^*fl/fl*^ mice in the presence and absence of MTP inhibitor. We found moderate TAG transfer activity in adipose tissue homogenate, which was significantly inhibited by MTP inhibitor (Fig. 6E). We measured much less PL activity in adipose tissue, and this was not inhibited by lomitapide (Fig. 6F) indicating that our assay may not be sensitive to measure PL transfer activity in tissues that express very low amounts of MTP.

### Recommended assay conditions to measure the PL transfer activity of MTPs in cells and tissue homogenates

There is a possibility of spontaneous transfer of NBD-PL between DV and AV that may increase background fluorescence. To avoid this higher background, we store DV and AV in separate tubes. For optimal assay conditions, we recommend using DV containing NBD-DOPE + unlabeled DOPE in PC vesicles as follows. Just before the assay, equal volumes of both vesicles (AV + DV) are mixed, and 10 μl of this vesicle mixture is added to the wells of a black/opaque plate. Then 90 μl containing the MTP source is added, and NBD fluorescence is measured. When measuring the PL transfer activity of MTP in cell and tissue homogenates, proper protein concentrations must be used. At higher concentrations, other PL transfer proteins may interfere with MTP transfer activity, whereas at lower concentrations, the MTP activity may not be sufficiently robust relative to the blank signal. We observed that 30–50 μg of protein was appropriate for measuring the PL transfer activity of MTPs in Cos-7 cells expressing hMTP, Huh-7 cells, and mouse liver tissue. Depending on tissue type and MTP abundance, the optimal protein concentration for the assay should be determined. When high protein concentrations are used, background autofluorescence should be measured in parallel wells. We also observed that the assay performs better at room temperature (25 ± 2°C) than at 37°C. With 80 ng of purified hMTP, we observed 20 ± 5% PL transfer in 4 h. The intra-assay %CV was 10 (n = 9), and the interassay coefficient was 18 (n = 5).

### Schematic depiction of the lipid transfer assay

**Fig. 7:**
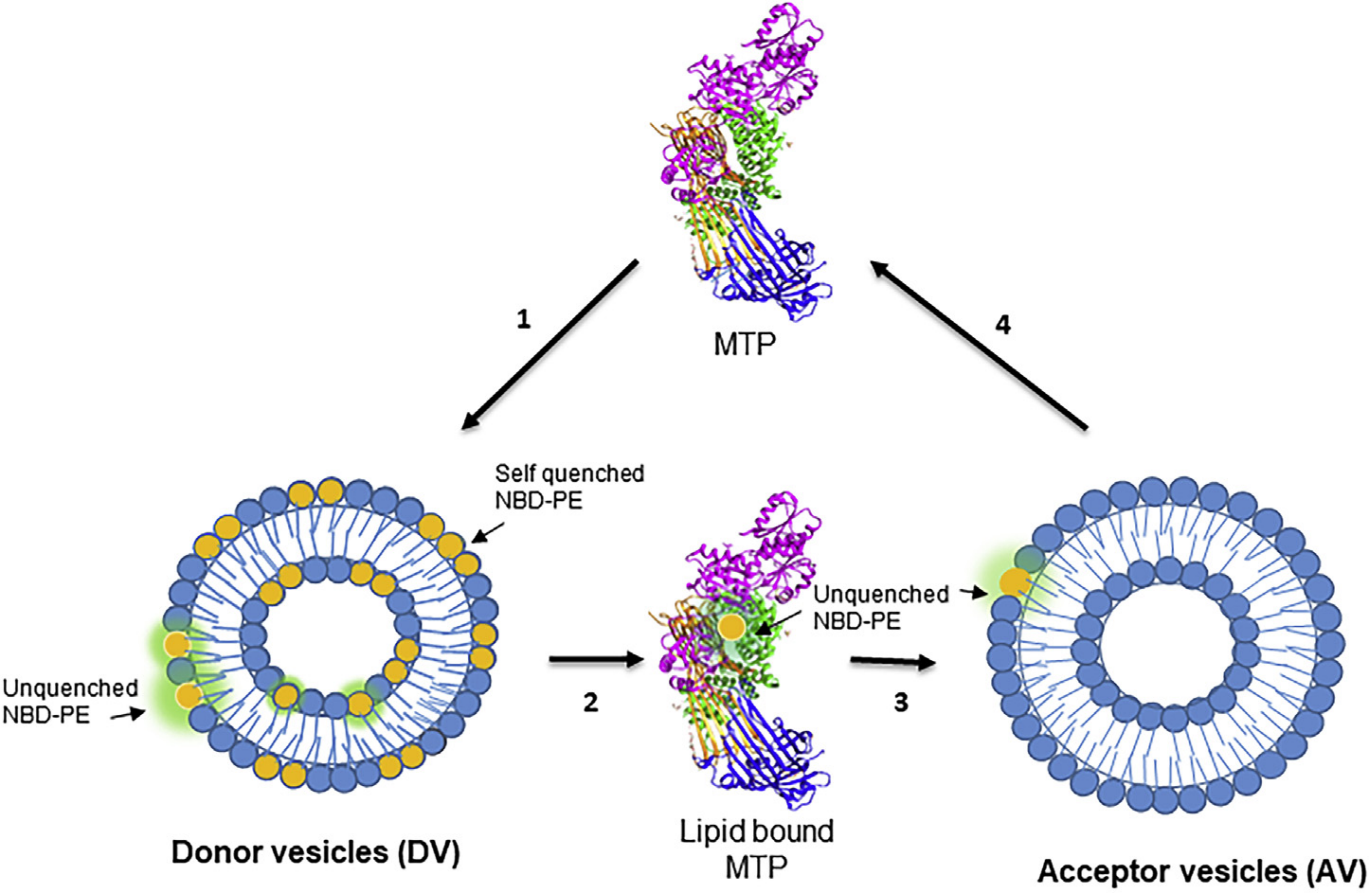
Schematic depiction of the PL transfer assay of MTP. In a typical transfer assay, AV and DV are incubated with a source of MTP. The MTP transfers lipids between these vesicles. DV contain both quenched (yellow) and unquenched (yellow with green cloud) fluorescent PE, as indicated. We envision different steps in the transfer of lipids by MTP. *1*) MTP interacts with DV. *2*) MTP picks up lipids from DV. During this process, the fluorescence in MTP-bound NBD-DOPE is probably unquenched (yellow with green cloud) and is detectable. *3*) MTP deposits the NBD-PE in AV. This deposited lipid is dispersed in the PC bilayer of AV and therefore remains unquenched. Measurements of this fluorescence represent the amount of lipid transferred. *4*) After depositing NBD-PE in AV, the MTP is again available for another cycle of lipid transfer. Repeated cycles of PL transfer from DV to AV by MTP contribute to time-dependent increases in fluorescence and represent MTP activity.

The schematic diagram (Fig. 7) attempts to explain different events in the transfer assay. The assay reaction mix contains DV, AV, and MTP. In the first step, MTP transiently binds DV and extracts the self-quenched NBD-PE. After extraction from the vesicle bilayers, MTP-bound NBD-PE is no longer quenched and therefore emits fluorescence. In the third step, MTP deposits the NBD-DOPE in AV, in which the NBD-DOPE remains dispersed and fluorescent. In this assay, the AV PC concentration is 7.5-fold greater than that in DV, and hence the chance of two or more transferred NBD-PE molecules coming together and becoming self-quenched is low. Therefore, the transferred NBD-PE continuously emits fluorescence, which is measured by a spectrofluorometer. The fluorescence intensity is proportional to the amount of PL transfer. In the fourth step, the MTP becomes unbound or free and is ready for next cycle of transfer.

## Discussion

MTP exhibits slower transfer activity toward PL than TAG (5, 6, 24). Furthermore, other PL transfer proteins exist in cells. Therefore, to measure the low PL activity of MTP and the even lower activity of MTP mutants in the presence of other PL transfer proteins, an easy and sensitive assay is needed. In prior studies, PL transfer activity has been measured with DV made with radiolabeled PE (4, 6, 27). Handling and disposal of radioactive material is hazardous. Moreover, measurement of time-dependent kinetics in many samples simultaneously is difficult. To address these assay shortcomings, our laboratory previously developed assays using fluorescently labeled lipids (23, 24). Our TAG transfer assay was rapid and sensitive and was able to measure this activity in cell lysates. However, the PL transfer measurements were less sensitive and required purification of MTP to reliably measure PL transfer activity (17). To overcome these hurdles, we optimized the lipid composition of DV to measure PL transfer activity of MTP in cell and tissue homogenates. We used AV with the same lipid composition as previously reported (23, 24). To identify a better substrate, we tested four different NBD-labeled lipids: DMPE, DPPE, POPE, and DOPE. Vesicles made with NBD-POPE and NBD-DOPE showed no changes in the blank samples but significant increases in fluorescence over time in MTP samples. Thus, our results showed that DVs containing POPE and DOPE are suitable to measure MTP activity. These PEs contain one and two oleic acids, respectively. An unsaturated fatty acid with C18 carbon atoms is likely to be a better substrate for MTP than saturated fatty acids with C14 (myristic acid) and C16 (palmitic acid) carbons present in DMPE and DPPE. The reason why DVs containing DPPE were not suitable for transfer assays, in contrast to those containing DMPE, remains unclear. More studies are needed to determine the reason for this marked differential behavior in the transfer assays.

A caveat of the assay is that the highest PL transfer was observed at room temperature rather than at the physiological temperature of 37°C, possibly because the greater fluidity of membrane lipids at higher temperatures might have increased the lateral diffusion of PL monomers. To verify that the majority of PL transfer in samples was due to MTP, we used lomitapide, which inhibits MTP activity. This PL transfer assay required longer incubation times, of 2–4 h, than those of the TAG transfer assay. The reasons for these differences are unclear. Unlabeled PC present in DV and AV is likely to compete with NBP-PE transfer, thus leading to slower transfer of NBD-DOPE.

In the presence of MTP inhibitor, the PL and TAG transfer activities were similarly inhibited, thus confirming that MTP, rather than other proteins in cell or tissue homogenates, was responsible for the PL and TAG transfer activity. With similar specific inhibitors, this assay might be adapted to measure the activity of other PL transfer proteins. In addition, the use of specific NBD-labeled PL may aid in developing specific assays.

In conclusion, we report a significantly improved sensitive assay to measure the PL transfer activity of MTP. This simple and easy assay involves few steps, can be performed at room temperature, and measures MTP activity in cell and tissue homogenates. The drawbacks of this assay are that it measures PL transfer better at room temperature than at 37°C, the vesicles are stable for only 30 days, and a time of 2–4 h is required to resolve the activity above background. This assay robustly allows MTP activity to be easily assessed in several samples simultaneously. With possible automation, this assay would be suitable for high-throughput screening to identify antagonists and possibly activators, and identifying gain-of-function mutations.

## Data availability

All the data are contained in the article.

## Conflict of interest

The contents of this article do not represent the views of the Department of Veterans Affairs or the US Government. The authors declare that they have no conflicts of interest with the contents of this article.
